# Topical application of adipose tissue-derived mesenchymal stem cells (ADMSCs) reduced cerebral edema in experimental traumatic brain injury (TBI)—a preliminary study

**DOI:** 10.1186/s41016-020-00219-9

**Published:** 2021-01-04

**Authors:** Hui Ma, Lian Xu Cui, Ping Kuen Lam, Cindy S. W. Tong, Kin K. Y. Lo, George K. C. Wong, Wai Sang Poon

**Affiliations:** 1 Division of Neurosurgery, Chow Tai Fook-Cheng Yu Tung Surgical Stem Cell Research Center, Department of Surgery, The Chinese University of Hong Kong, Shatin, Hong Kong, SAR China; 2 Division of Neurosurgery, Department of Surgery, Fo Shan First People’s Hospital, Foshan, Guangdong China

**Keywords:** Topical, MSCs, Cerebral edema, TBI

## Abstract

**Background:**

Our previous studies showed that topical application of mesenchymal stem cells (MSCs) improved functional recovery in rat traumatic brain injury (TBI) model, and hypoxic precondition further enhanced the therapeutic effects of MSCs. There was no previous study on the attenuation of cerebral edema by MSCs.

We investigated whether topical application of normoxic and hypoxic MSCs could reduce cerebral edema in an experimental TBI model.

**Methods:**

Two million normoxic (*N* = 24) and hypoxic (*N* = 24) MSCs were applied topically to exposed the cerebral cortex in a controlled cortical impact (CCI) model. The MSCs were fixed in position with fibrin glue. No treatment was given to control animals (TBI only: *n* = 24). After surgery, four animals in each group were sacrificed daily (day 1 to day 6) for edema evaluation. Normal animals without TBI were used as reference (*n* = 4). The expressions of GFAP, AQP4, and MMP9 were also investigated by immunofluorescence staining and RT-PCR at day 3.

**Results:**

The edema peaked within 3 days after TBI. Compared with the control, hypoxic MSCs reduced brain water content significantly (*p* < 0.05). Both hypoxic and normoxic MSCs downregulated the expression of MMP9 and normalized AQP4 distribution to astrocyte end feet.

**Conclusion:**

Our preliminary study showed that topical application of hypoxic MSCs suppressed both vasogenic and cytotoxic edema formation.

## Background

Brain edema was defined as “an expansion of brain volume which increases intracranial pressure’, impairs cerebral perfusion, and causes additional ischemic injuries” [1]. For decades, edema has been a target in the treatment of traumatic brain injury. Conventional managements to reduce cerebral edema include (i) general measures: optimal head and neck position, adequate oxygenation, maintenance of normotension, management of fever and hyperglycemia, and nutritional support; (ii) specific interventions: controlled hyperventilation, osmotherapy (mannitol, hypertonic saline), corticosteroid administration, and pharmacological coma (barbiturates); and (iii) surgical procedures: cerebrospinal fluid (CSF) drainage, decompressive craniectomy, and decompressive laparotomy [2]. However, these passive treatments can induce complications such as renal failure and acute respiratory distress syndrome [2]. Classically, there are two types of edema associated with traumatic brain injury, namely vasogenic edema and cytotoxic/cell edema [3, 4]. Vasogenic edema is caused by disruption of blood-brain barrier (BBB) after TBI, resulting in water movement from the blood vessels to extracellular space and an increase in brain water content. Cytotoxic edema, also known as cellular or ionic edema, is defined as “cellular failure with disrupted ionic pump with anaerobic metabolism” which is caused by ischemia after TBI [5].

Mesenchymal stem cells (MSCs) are able to self-renewal and differentiate into multiple cell lineages. To accelerate functional recovery, MSCs bio-modulate the local microenvironment, suppress inflammatory response, and promote angiogenesis and regeneration in addition to the trans-differentiation after transplantation [6]. Our recent study showed that hypoxic preconditioning of MSCs enhanced tissue repair and functional recovery in experimental TBI [7]. To our knowledge, there are no reports on the attenuation of cerebral edema by MSCs.

We would like to investigate whether topical application of MSCs could reduce cerebral edema in a rodent model of TBI.

## Methods

**Fig. 1:**
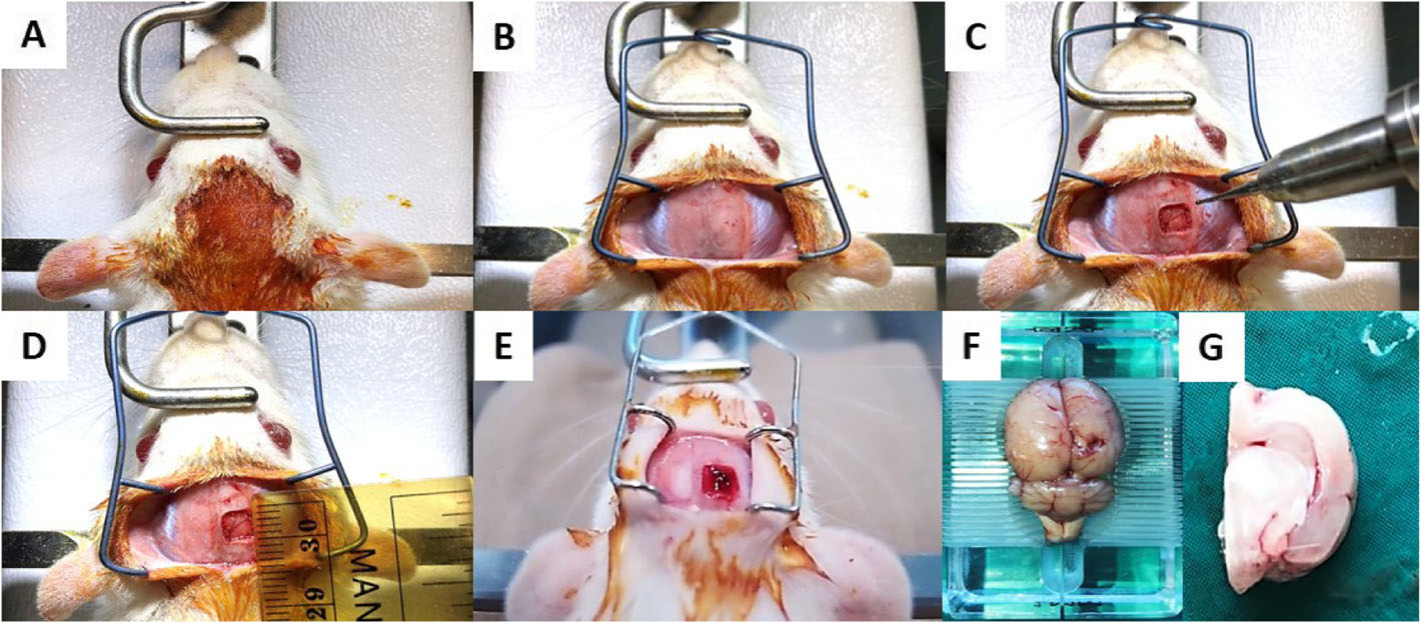
The process of controlled cortical injury (CCI) and injured hemisphere collection. **a**–**e** Process of CCI model on SD rat. **f** Right hemisphere was injured. **g** The hemisphere harvested for brain water content measurement

MSCs were derived from the subcutaneous adipose tissue of male SD rats. Cultured under either normoxic (18% O_2_) or hypoxic (5% O_2_), all MSCs expressed CD29 and CD90 but not CD45. The MSCs had adipogenic, chondrogenic, and osteogenic differentiation potentials in vitro under specific culture conditions. Female SD rats, aging 10–12 weeks were used for the study (Laboratory Animal Services Center, CUHK). Traumatic brain injury was induced by controlled cortical impact (CCI) [7] (Fig. 1). After surgery, 2 million normoxic (*n* = 24) and hypoxic (*n* = 24) MSCs were applied topically to the exposed brain cortex. A thin layer of fibrin glue was used to keep cells in position. No treatment was given to animals in the control group (*n* = 24) (Table 1).

**Table 1:** Animal groupings in the study

Groupings	Number of animals	Treatment
Control	24	TBI without treatment
MSCs	24	TBI + 2 million MSCs
Hypoxic MSCs	24	TBI + 2 million hypoxic MSCs

Four animals in each group were sacrificed daily until day 6, and normal animals were used as reference (*n* = 4). For each animal, the injured hemisphere was collected and dried in the oven for 36 h. Wet weight and dry weight were measured respectively. Brain water content was calculated according to the formula: (wet weight-dry weight)/wet weight %.

Hydrated paraffin tissue slices were stained by anti-GFAP (Abcam, Cambridge, UK) (a marker of astrocytes) and anti-AQP4 (Abcam, Cambridge, UK) (a marker of water channel on astrocytes) antibody. Real-time PCR was used for the determination of MMP9 (Abcam, Cambridge, UK) (marker of vasogenic edema) expression.

## Results

### Hypoxic MSCs reduced brain water content

**Fig. 2:**
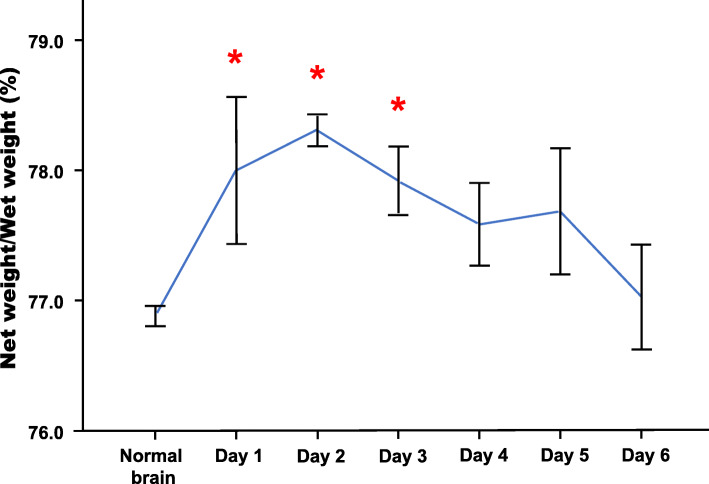
4 animals were sacrificed daily until day 6 after TBI. The injured hemisphere was harvested and measure brain water content. Compared with hemisphere from normal animals, the brain water content was significantly higher from day 1 to day 3 (*p < 0.05 vs control) (net weight = wet weight-dry weight)

**Fig. 3:**
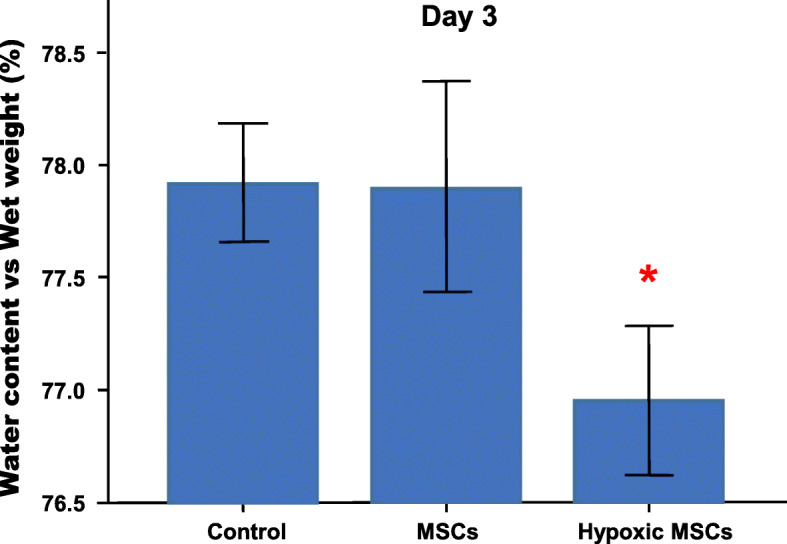
The injured hemispheres were harvested from animals in different groups on day 3. The hypoxic MSC-treated brain showed lower water content than control (*p < 0.05 vs control), and no remarkable change was found between normoxic MSC-treated brain and control

The brain water content increased remarkably within 3 days after TBI (Fig. 2) (Table 2). Then, it reduced significantly in the hypoxic MSC group (*p* < 0.05 vs control), whereas no significant change was found in the normoxic group (Fig. 3).

**Table 2:** The average wet and dry weight of the injured hemisphere

Groups/time point	Wet weight (g)	Dry weight (g)	Water content (%)
Normal	0.73	0.17	76.88
Control-D1	0.73	0.16	78.00*
Control-D2	0.68	0.15	78.31*
Control-D3	0.70	0.15	77.92*
Control-D4	0.64	0.14	77.59
Control-D5	0.67	0.15	77.69
Control-D6	0.67	0.15	77.03
MSCs-D3	0.63	0.14	77.90
Hypoxic MSCs-D3	0.70	0.16	76.95^#^

^*^p < 0.05 vs normal

^#^*p* < 0.05 vs control

### Hypoxic MSCs normalized the AQP4 distribution

**Fig. 4:**
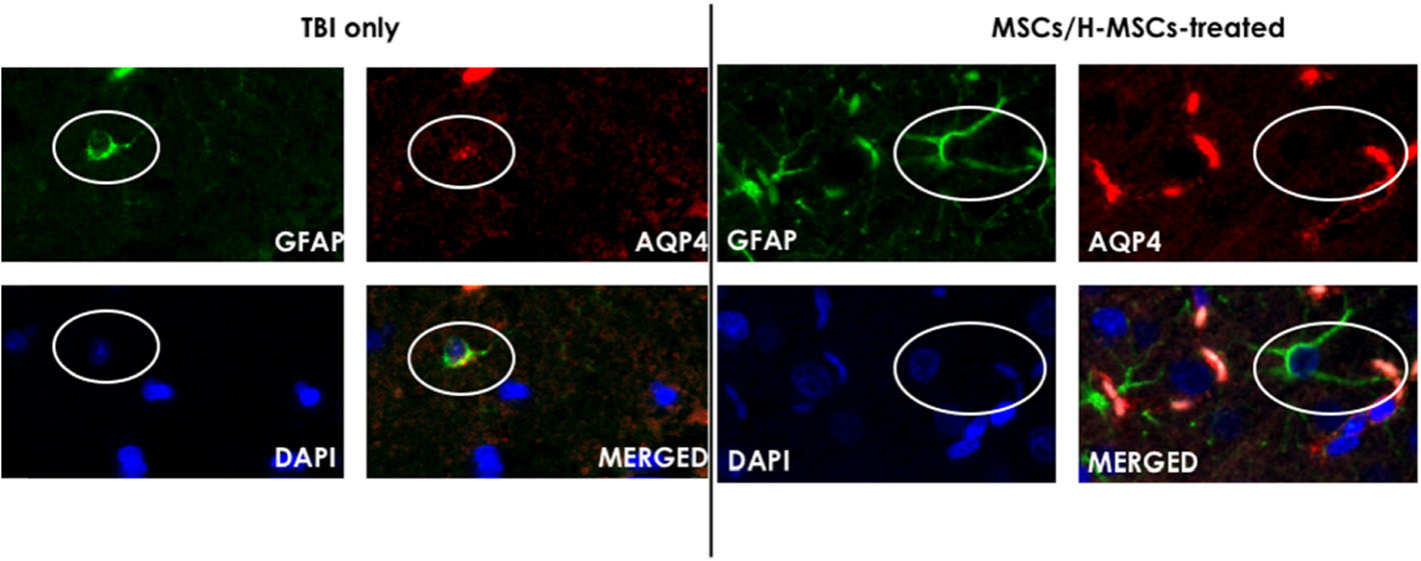
Picture of double immune-florescent staining of GFAP and AQP4. After TBI, distribution of AQP4 signal spread to cell body of astrocytes. With treatment of normoxic/hypoxic MSCs, the signal located to end feet of astrocytes

Double fluorescent staining of GFAP and AQP4 indicated that AQP4 distribution was normalized to the end feet of astrocytes after either normoxic or hypoxic MSC treatment, whereas a positive fluorescent signal was observed around the cell body of astrocyte in the control group (Fig. 4).

### Hypoxic MSCs downregulated the expression of MMP-9

**Fig. 5:**
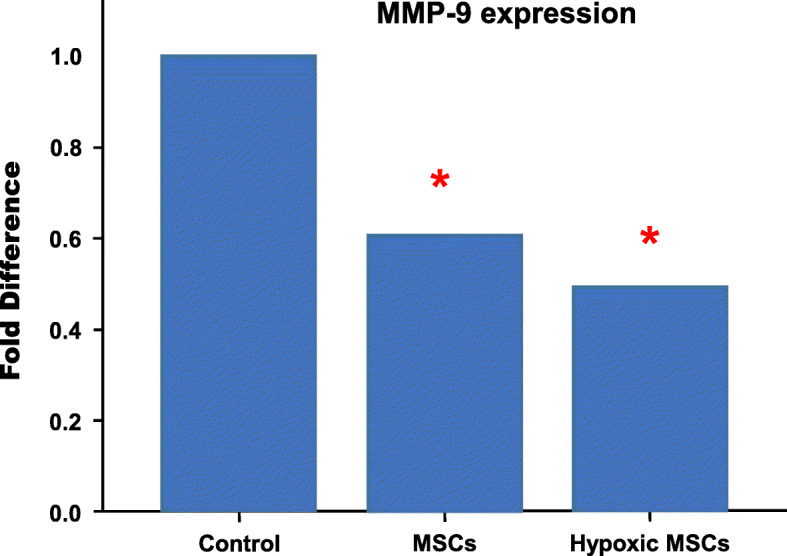
RT-PCR results indicated MMP9 expression was downregulated significantly after both normoxic and hypoxic MSC transplantation (*p < 0.05 vs control). No statistical difference was found between two cell treatments

Expression of MMP9 was downregulated significantly in both normoxic (*p* < 0.05 vs control) and hypoxic (*p* < 0.05 vs control) groups on day 3. No significant difference was found between two different MSC groups (Fig. 5) (Table 3).

**Table 3:** List of primers used in RT-PCR

GAPDH	Forward AGA CAG CCG CAT CTT GT
	Reverse CTT GCC GTG GGT AGA GTC AT
MMP-9	Forward CCC CAC TTA CTT TGG AAA CGC
	Reverse ACC CAC GAC GAT ACA GAT GCTG

## Discussion

The pathophysiology of brain edema involves vasogenic and cytotoxic cascades. Vasogenic edema is associated with blood-brain barrier (BBB) integrity. Also, brain water content increases due to BBB dysfunction. In this study, we demonstrated that topical application of hypoxic MSCs reduced brain water content significantly on day 3. This effect was not observed in the normoxic MSC treatment group. Previous study showed hypoxic preconditioning enhanced therapeutic effects of MSCs [7]. Thus, it was believed that the suppression effects against cerebral edema were stronger in the hypoxic MSC group. Animal studies showed the upregulation of MMP9 in TBI [8, 9]. MMP9 degrades extracellular matrix proteins, including neurovascular basal lamina and tight junction proteins of BBB [10]. The downregulation of MMP9 by MSC treatment could reduce edema formation and maintain BBB integrity [10]. AQP4, one of the water channel proteins, is a key factor in the development and resolution of cerebral edema [11]. Normally, it locates in the perivascular astrocyte end feet [12]. After traumatic brain injury occurs, astrocytosis is activated, and the AQP4 signal is not limited to the end feet. It is also detected all reactive astrocytes [12]. Topical application of MSCs suppressed neuro-inflammation by triggering reactive astrocytosis at the early phase [6]. In this study, topical MSCs normalized AQP4 distribution in astrocytes. In the tissue without MSC treatment, the astrocytes underwent swelling after TBI, resulting in the opening and re-location of the water channel on the cell body. The topically applied MSCs contributed to the suppression of cerebral edema and the reduction of astrocytes swollen, shown as the normalization of the AQP4 distribution. Although the underlying mechanism is not yet clear, there are two hypotheses to explain the attenuation of cerebral edema. Firstly, numerous studies have shown that the transplanted MSCs were capable to express a marker of astrocytes (GFAP) in vivo, which may help maintain BBB integrity [13]. Secondly, MSCs secrete soluble factors including growth factors, cytokines, and chemokines, through paracrine activity [13]; they might biomodulate the neuro-inflammation associated with cerebral edema.

However, we have some limitations in this study. First, we did not measure brain water content at the acute phase after TBI, such as 1 h, 6 h, and 12 h. Second, we did not compare therapeutic effects of normoxic and hypoxic MSCs in the acute phase. Third, we compare water content only in the injured hemisphere instead of the whole brain.

## Conclusion

In this study, we demonstrated topical application could deliver a large amount of MSCs safely and effectively to the injured brain. Hypoxic MSCs reduced brain water content 3 days after the injury happened, both normoxic and hypoxic MSC treatment downregulated pro-vasogenic edema gene expression and normalize water channel distribution in astrocytes.

## Data Availability

Please contact the corresponding author if any data or materials are required.

## Abbreviations

ADMSCs: Adipose tissue-derived mesenchymal stem cells
BBB: Blood-brain barrier
CCI: Controlled cortical impact
CSF: Cerebrospinal fluid
MSCs: Mesenchymal stem cells
TBI: Traumatic brain injury
